# Comprehensive insights on pivotal prognostic signature involved in clear cell renal cell carcinoma microenvironment using the ESTIMATE algorithm

**DOI:** 10.1002/cam4.2983

**Published:** 2020-04-20

**Authors:** Jun Luo, Yi Xie, Yuxiao Zheng, Chenji Wang, Feng Qi, Jiateng Hu, Yaoting Xu

**Affiliations:** ^1^ Department of Urology Shanghai Fourth People's Hospital affiliated to Tongji University School of Medicine Shanghai China; ^2^ The First Clinical Medical College of Nanjing Medical University Nanjing China; ^3^ Department of Urology Jiangsu Cancer Hospital Jiangsu Institute of Cancer Research Nanjing Medical University Nanjing China; ^4^ State Key Laboratory of Genetic Engineering Collaborative Innovation Center for Genetics and Development School of Life Sciences Fudan University Shanghai China; ^5^ Department of Urology The First Affiliated Hospital of Nanjing Medical University Nanjing China

**Keywords:** biomarkers, clear cell renal cell carcinoma (ccRCC), immune infiltrates, immune/stromal scores, tumor microenvironment (TME)

## Abstract

Emerging evidence has highlighted that the immune and stromal cells formed the majority of tumor microenvironment (TME) which are served as important roles in tumor progression. In our study, we aimed to screen vital prognostic signature associated with TME in clear cell renal cell carcinoma (ccRCC). We obtained total 611 samples from TCGA database consisting of transcriptome profiles and clinical data. ESTIMATE algorithm was applied to estimate the infiltrating fractions of immune/stromal cells. We found that the immune scores revealed more prognostic significance in overall survival and positive associations with risk clinical factors than stromal scores. We carried out differential expression analysis between Immunescore and stromalscore groups to obtain the 72 intersect genes. Protein to protein interaction (PPI) network and functional analysis was performed to indicate potential altered pathways. Additionally, we further conducted multivariate Cox analysis to identify 12 hub genes associated highly with TME of ccRCC using a stepwise regression procedure. Accordingly, risk score was constructed from the multivariate Cox results and Receiver Operating Characteristic (ROC) curve was used to assess the predictive value (AUC = 0.781). The ccRCC patients with high risk scores suffered poor survival outcomes than that with low risk scores. In the validation cohort from GSE53757, TNFSF13B, CASP5, and GJB6 correlated positively with tumor stages, while FREM1 negatively correlated with tumor stages. Importantly, we further observed that TNFSF13B, CASP5 and XCR1 showed the remarkable correlations with tumor‐infiltrating immune cells. Taken together, our research identified specific signatures that related to the infiltration of stromal and immune cells in TME of ccRCC using the transciptome profiles, which reached a comprehensive understanding of tumor microenvironment in ccRCC.

## Graphical abstract

Our research identified specific signatures related to the tumor microenvironment (TME) of ccRCC using the transcriptome profiles. Besides, we further discussed the associations of TME‐related signature with tumor‐infiltrating immune cells, which shed light on a comprehensive understanding of TME and future individualized therapy.

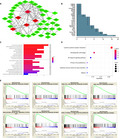

## 1 INTRODUCTION

Renal cell carcinoma (RCC), accounting for more than 90% of kidney malignancies and comprising almost 2%‐3% among all human malignant neoplasms, is the second most common malignancy in the urinary system second to bladder cancer.^1^, ^2^, ^3^ The incidence and mortality of RCC have increased rapidly in recent decades. In 2019, the estimated new cases and new death of kidney cancer will increase to 73 820 in the United State.^2^ As the most common histologic subtype of RCC according to pathologic classification, clear cell renal cell carcinoma (ccRCC) accounts for approximately 70% of all RCC cases.^4^, ^5^ Studies have shown that the tumorigenesis and development of ccRCC is a complex progress mediated by various drivers, environmental risk factors such as obesity and smoking, or tumor microenvironment (TME) alterations.^6^, ^7^, ^8^, ^9^ However, the molecular regulation mechanisms of ccRCC tumorigenesis and progression is still unclear.

TME is the complex cellular milieu containing immune cells, mesenchymal cells, endothelial cells, inflammatory mediators and extracellular matrix molecules adaptively or innately.^10^, ^11^, ^12^ To provide a comprehensive view of TME, PhenoGraph clustering algorithm were performed by Chevrier et al and the classification results indicate that T cells, with a mean of 51%, act as a key character in ccRCC TME immune cells. Besides, the proportion of myeloid cells, natural killer cells and B cells were 31%, 9%, and 4%, respectively.^9^, ^13^, ^14^ Previous studies often focused on the malignant progression of tumors regulated by some particular types of non‐tumor cells or regulators in TME. There is a limitation of comprehensive studies analyzing the prognostic value of TME in malignant tumors from a genome‐wide perspective.

In recent years, the establishment of public resources and the emergence of new biological algorithms have provided new data resources and technical means for TME research. The Cancer Genome Atlas (TCGA) database is a public data resource consisting of cancer‐causing genomic alterations among various malignancies.^15^ Moreover, the Gene Expression Omnibus (GEO) database with biological information from the National Center for Biotechnology Information (NCBI) provides a promising approach for extracting high‐through sequence information.^16^ Novel algorithms have been invented to evaluate tumor purity according to TCGA database.^17^, ^18^ Yoshihara et al described a new algorithm called “Estimation of STromal and Immune cells in MAlignant Tumours using Expression data” (ESTIMATE), which is capable of calculating the fraction of different cells in malignant tumors utilizing gene expression signatures.^17^ Since the infiltration levels of normal cells in tumor microenvironment also function a significant role in tumor progression, we mainly utilized the unique properties of the transcriptional traits to assess the cellularity of various infiltrating normal cells, including the two main types of stromal and immune cells. The utility of ESTIMATE algorithm was widely reported to successfully predict the infiltration of nontumor cells in TMEs of prostate cancer, breast cancer and colon cancer.^19^, ^20^, ^21^ However, limited research explored the TME of ccRCC adopting ESTIMATE algorithm.

In the present study, ESTIMATE algorithm was firstly performed to calculate the immune and stromal scores of TME in ccRCC. We extracted the high‐throughput sequencing data of ccRCC and identified pivotal genes associated with TME of ccRCC. Importantly, we established a corresponding risk score system to predict the survival outcomes of ccRCC patients, and further explored the underlying relationships between TME‐related signature and immune infiltrates.

## 2 MATERIALS AND METHODS

### 2.1 Data collection and processing

We obtained the RNA‐seq data (Level 3) of TCGA‐KIRC cohort (https://portal.gdc.cancer.gov/), including 539 ccRCC and 72 normal samples. Corresponding clinical characteristics of age, gender, tumor grade, pathological stage, AJCC‐TNM, and survival outcomes were downloaded from TCGA portal using the GDC tool. We utilized the limma package to conduct the normalization process, deleting the normal or repeated samples for subsequent analysis.

ESTIMATE algorithm was exploited to infer the fraction of immune and stromal cells in tumor tissues based on gene expression signature, including microarray expression data sets, new microarray, as well as RNA‐seq transcriptome profiles. We downloaded the R script of ESTIMATE algorithm from the public source website (https://sourceforge.net/projects/estimateproject/). Then, we calculated the immune scores, stromal scores and ESTIMATE scores for each sample, respectively (Table S1).

### 2.2 Survival analysis and correlation analysis

We utilized the survival package to conduct the Kaplan‐Meier analysis for ccRCC patients based on the immune scores, stromal scores and ESTIMATE scores. The respective *P* value of the log‐rank test was calculated and considered as significant with *P* < .05. Meanwhile, we further assessed the associations between score levels and multiple subgroups of clinical variables using Kruskal‐Wallis (W‐S) test, which was a nonparametric test suitable for comparisons among two or more groups. *P* < .05 was thought to be of statistical significance.

### 2.3 Differentially expressed genes and clustering analysis

Since we obtained three scores from the ESTIMATE method, we could classify the samples into high‐ and low‐level groups according to the median score, respectively. For two groups of immune scores, we used the limma package to analyse the transcriptome data with |log(FC)| > 1 and False Discovery Rate (FDR) < 0.05 as the threshold.^22^ Meanwhile, we conducted the clustering analysis to identify significant up and down gene sets between the two immune score levels and illustrated the differential genes using pheatmap package. Accordingly, we performed the same procedure and differential analysis in patients with high‐ and low‐level stromal scores. Furthermore, we identified the intersect genes of four gene sets from the differential analysis of patients with immune scores and stromal scores. VennDiagram package was exploited to visualize the process and intersect genes (Figure 3B).^23^

### 2.4 PPI network and pathway analysis, Gene Set Enrichment Analysis (GSEA)

Intersect genes were selected as the vital genes associated with tumor microenvironment. We utilized the STRING database to construct the protein‐protein interaction (PPI) network and modified the plot using Cytoscape software (version 3.7.1) based on JAVA8.0 platform.^24^, ^25^ Besides, we calculated the number of connecting nodes for top 30 genes and shown the results in barplot. What is more, we further investigate the potential biological pathways that intersect genes may participate in. Firstly, we exploited the org.Hs.eg.db package to obtain the entrez ID of each genes. Then, clusterProfiler, org.Hs.eg.db, enrichplot, and ggplot2 packages were utilized to perform the gene ontology analysis from three aspects consisting of Cellular Component (CC), Molecular Function (MF), and Biological Process (BP), which was illustrated by barplot. In addition, Kyoto Encyclopedia of Genes and Genomes (KEGG) was performed to conduct the pathway analysis, which was shown by dotplot.^26^ The *P* value <.05 were considered to be significant.

We downloaded the GSEA software (http://software.broadinstitute.org/gsea/index.jsp), running based on JAVA8 platform.^27^ Then, we selected the immune scores as the phenotypes and divided the samples into high‐ and low‐groups. Afterwards, “c2.cp.kegg.v6.2.symbols.gmt gene sets” was chosen from the MSigDB (http://software.broadinstitute.org/gsea/downloads.jsp) to be used as the reference gene sets. Last, *P* < .05 was considered statistically significant.

### 2.5 Establishment of risk score

To further identify important genes in tumor microenvironment, we conducted the Kaplan‐Meier analysis to select prognostic genes with *P* value of log‐rank test <.05. We mainly used the survival package and “for cycle” R script to conduct the survival analysis of all genes. Then, we performed the stepwise regression method to screen 12 hub prognostic genes associated with ccRCC microenvironment, in which the minimum Akaike information criterion (AIC) value was obtained. Meanwhile, we used the multivariate Cox regression analysis to get the coefficients (β_i_) of each gene and calculated the risk score as following: risk score = Ʃ (β_i_ * Exp_i_) (i = 12). We draw the forest plot by survminer package to show the hazard ratio (HR) with 95% confidence interval (CI) of each gene. In addition, we could divide the ccRCC patients into high‐ and low‐risk groups according the median data of risk scores. We conducted the receiver operating characteristic curve (ROC) to assess the predictive value of risk score by survivalROC package.^28^ Kaplan‐Meier analysis was conducted to analysis the survival difference between high‐ and low‐risk group by survival package.

We also obtained 72 ccRCC patients from GSE53757 with transcriptome chip data and corresponding clinical stage information. We validated the 12 hub genes in the GSE53757 populations. The expression data of 12 genes were extracted and the risk score was calculated as the above formula. Kruskal‐Wallis test was utilized to evaluate the associations between expression levels of genes with clinical tumor stage.

### 2.6 TIMER database analysis

The TIMER database (https://cistrome.shinyapps.io/timer/) is a publicly available resource to estimate the abundance of tumor immune infiltrates using the deconvolution algorithm, including 10 897 samples across 32 types of cancers from TCGA. We invented to explore the associations between 12 hub genes with key immune infiltration cells, including B cell, CD4^＋^ T cell, CD8^＋^ T cell, macrophage, neutrophil, dendritic cell, as well as tumor purity. The Person’s correlation coefficients with corresponding statistical significance were calculated. We displayed the log2 (RSEM) of gene expression level in y‐axis.

### 2.7 Statistical analysis

Univariate Cox regression, multivariate Cox regression analysis and Kaplan‐Meier analysis were conducted by survival package. Differential analysis was performed by limma package. Kruskal‐Wallis (W‐S) test was mainly used for comparisons across two or more groups. The correlation of gene expression levels with immune infiltrates were determined by Pearson's coefficients combined with estimated statistical significance. All statistical analysis was conducted using R software (version 3.5.2). The *P* value <.05 was regarded to be statistically significant.

## 3 RESULTS

### 3.1 Immune scores revealed more prognostic value in ccRCC versus stromal scores

**FIGURE 1:**
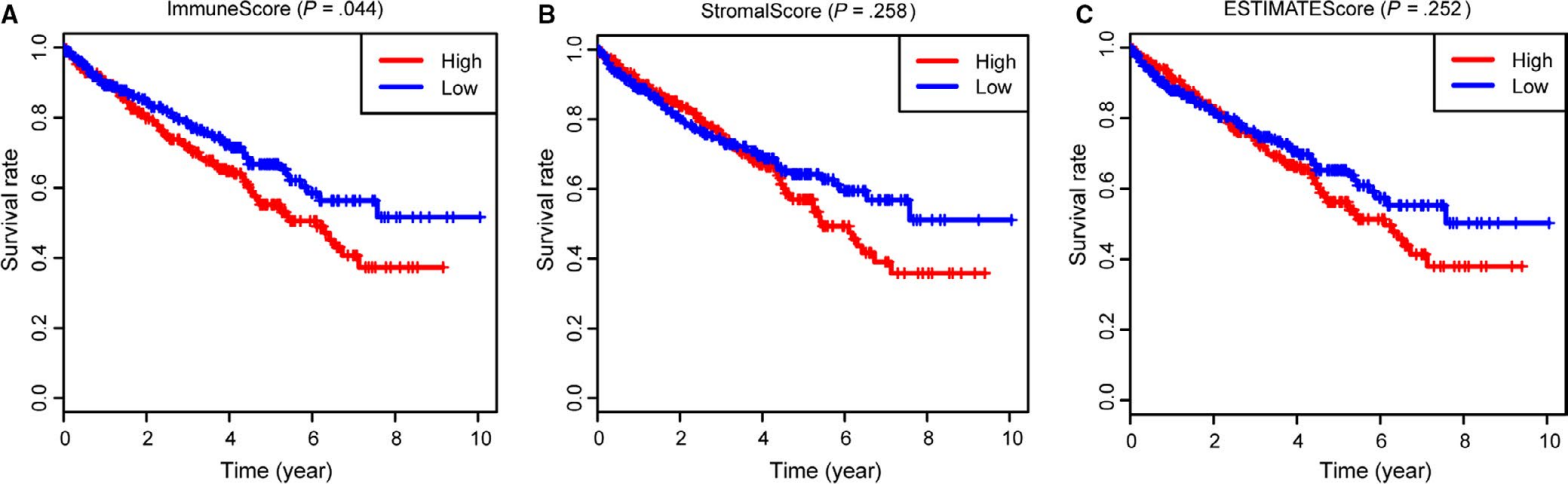
Survival analysis of immune scores, stromal scores and ESTIMATE scores with overall survival (OS). A, Clear cell renal cell carcinoma patients were divided into high group (n = 270) and low group (n = 269). As shown in Kaplan‐Meier plot, median survival of patients in the high group was shorter than that in low group indicated by the log‐rank test of *P* = .044. B, Similarly, no significant difference were observed in survival outcomes in patients with high‐ and low‐stromal scores (*P* = .258). C, There was no statistical prognostic difference in patients with high‐ an low‐ESTIMATE scores (*P* = .252)

We obtained a total of 611 samples from TCGA‐KIRC combined with transcriptome data and clinical information. Excluding nine cases with incomplete data or 72 normal samples, we got 530 ccRCC patients, in which 344 cases were male and 186 were female. The percentage of other clinical features was shown in Table 1. Moreover, the information of other patients from GSE53737 was shown in Table 2. We conducted the ESTIMATE method to calculate the immune scores, stromal scores and ESTIMATE scores for each sample, respectively. Immune scores ranged from −693.96 to 3328.21, while the distribution of stromal scores was −1433.77 to 1967.19 (Table S1). The ESTIMATEScore was calculated by integrating the two scores and the mean was 2283.99 ranging from −2127.72 to 5091.59. We then conducted the survival analysis to assess the prognostic value of the two scores. The log‐rank test revealed that only immune scores showed the statistical difference, where ccRCC patients with high immune scores correlated with poor survival outcomes (Figure 1; *P* = .044). However, no significant difference was observed in the stromal scores (*P* = .258), or the sum ESTIMATE scores (Figure 1; *P* = .252). Moreover, we divided the patients into three groups incorporating high, median with low groups, and the differential survival outcomes between high versus low subgroups could be seen in Figure S4.

**TABLE 1:** Clinical baseline of 530 ccRCC patients included in study from TCGA cohort

Variables	Number	Percentage
Vital status		
Alive	166	31.32
Dead	364	68.68
Age	60.56 ± 12.14	
Gender		
Female	186	35.10
Male	344	64.90
AJCC‐T		
T0/Ta	0	0
T1	271	51.13
T2	69	13.02
T3	179	33.77
T4	11	2.08
AJCC‐N		
N0	239	45.09
N1	16	3.02
NX	275	51.89
AJCC‐M		
G1/G2	241	45.47
G3/G4	286	53.96
Unknown	3	0.57
Stage		
Stage Ⅰ & Ⅱ	322	60.75
Stage Ⅲ & Ⅳ	208	39.25
Immune score		
Low level	265	50.00
High level	265	50.00
Stromal score		
Low level	265	50.00
High level	265	50.00
ESTIMATE score		
Low level	265	50.00
High level	265	50.00
Risk score		
Low level	265	50.38
High level	265	49.62

Abbreviations: AJCC, American Joint Committee on Cancer.

**TABLE 2:** Tumor stage of 72 ccRCC patients in GSE53737

Clinical stage	Samples (n)	Percentage
Stage Ⅰ	24	33.34
Stage Ⅱ	19	26.39
Stage Ⅲ	14	19.44
Stage Ⅳ	15	20.83

**FIGURE 2:**
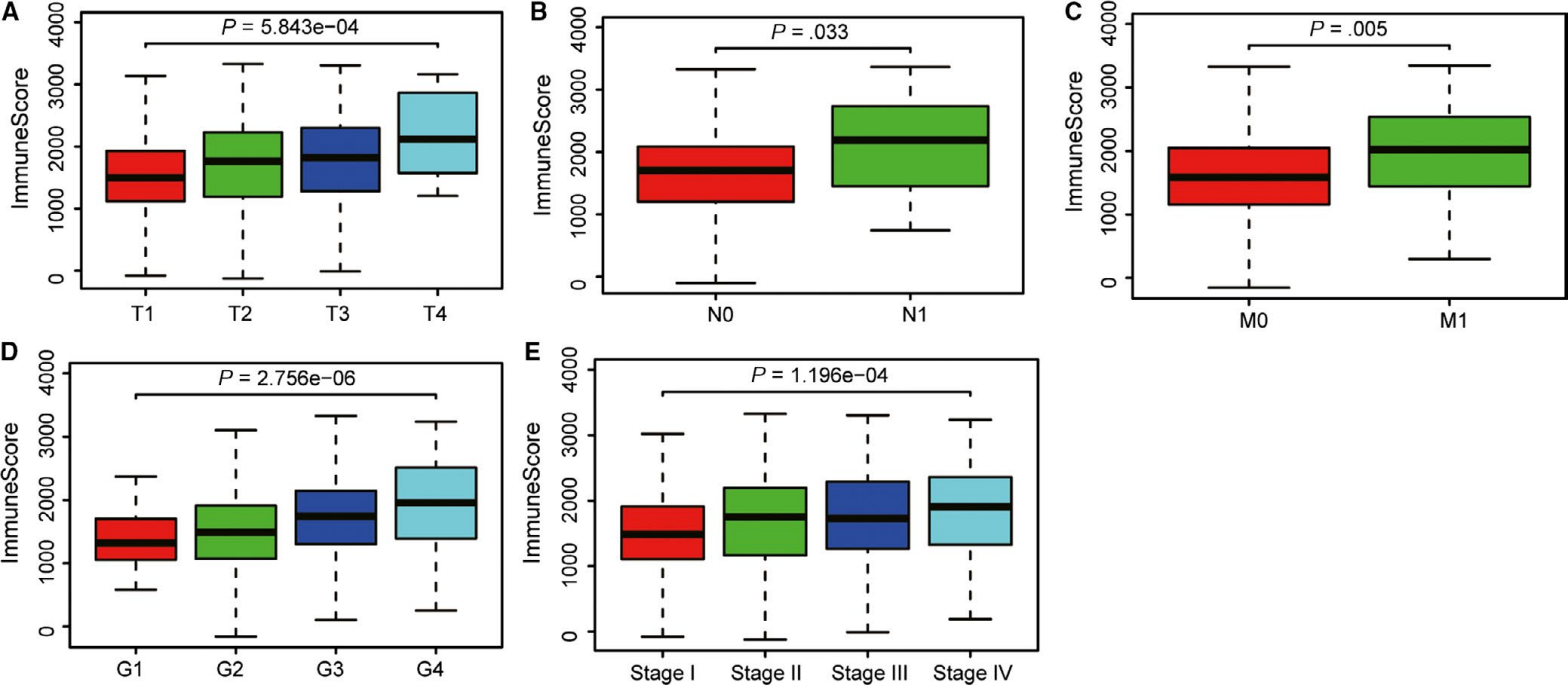
Correlation analysis of immune scores with risk clinical variables using Kruskal‐Wallis (W‐S) test. A‐C, Higher expression level of immune scores correlated with higher AJCC‐T stage, higher AJCC‐N stage, and advanced metastasis. D, Higher expression level of immune scores were associated with higher tumor grades. E, In addition, higher immune scores distributed in higher pathological stages

Additionally, we further investigated the immune scores and stromal scores with independent clinical characteristics, including tumor grade, pathological stage, and AJCC‐TNM stage. The Kruskal‐Wallis (W‐S) test revealed that immune scores were associated with higher AJCC‐T level (*P* < .001), higher AJCC‐N level (*P* < .05), higher AJCC‐M level (*P* < .001), advanced tumor grades (*P* < .001), as well as higher pathological stages (*P* < .001) (Figure 2). However, there were no significant difference among stromal scores with any other clinical features (Figure S1), in accordance with the results from above survival analysis.

### 3.2 Differential analysis of gene expression profiles with stromal scores and immune scores in ccRCC

**FIGURE 3:**
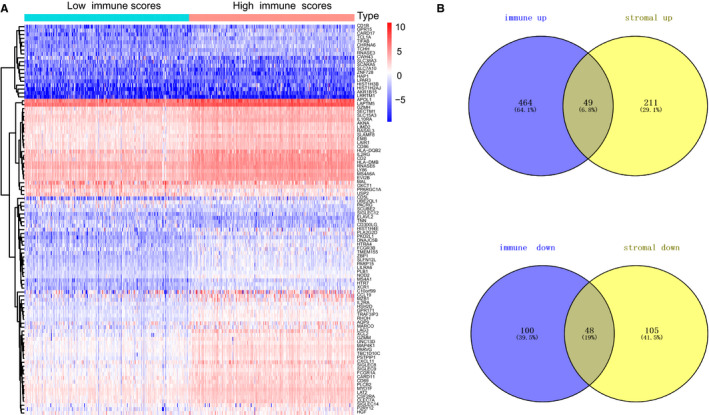
Differentially expressed genes analysis with immune scores in clear cell renal cell carcinoma. A, Heatmap of differentially expressed genes in two levels of immune scores was illustrated by pheatmap package with FDR < 0.05, fold change > 1. B, Identification of intersect genes of commonly up‐regulated and down‐regulated in stromal and immune scores. The number also displayed in the Venn diagrams

Limma package was mainly used to deal with Affymetrix microarray data of 539 ccRCC patients. For samples with immune scores, we classified the patients into high‐ (n = 270) and low‐level (n = 269) groups according the median immune scores. Heatmap in Figure 3A revealed the clustering analysis. We totally identified 659 differentially expressed genes (DEGs) based on immune scores, consisting of 512 highly expressed genes (fold‐change ＞ 1, FDR < 0.05) and 147 down‐regulated genes (fold‐change < −1, FDR < 0.05). Meanwhile, we divided the patients with stromal sores into high‐ (n = 270) and low‐level (n = 269) groups. The differential analysis was performed by the same process and 259 up‐regulated genes with 152 down‐regulated genes were identified (Table S2; Figure S2). To further identify vital genes associated with microenvironment, we exploited the Venn diagrams to search 97 intersect genes, where 49 genes were all up‐regulated in ccRCC samples with higher immune/stromal scores and 48 genes were all down‐regulated in that with lower immune/stromal scores. Last, when clustering the samples, the differential genes still significantly discriminate patients of low and high scores in Figure S5.

### 3.3 Protein‐protein interactions among intersect genes and functional enrichment analysis

**FIGURE 4:**
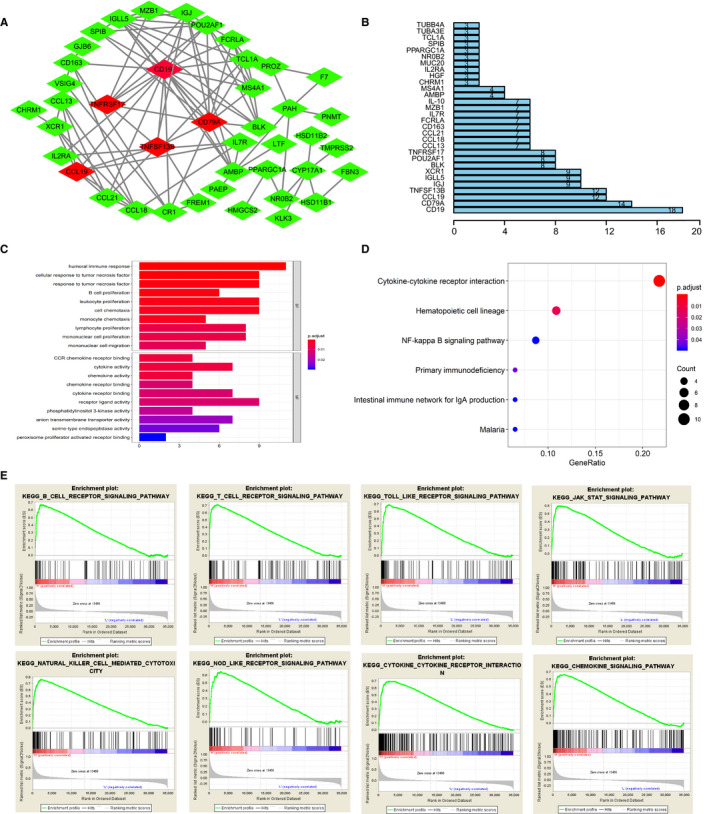
Construction of protein to protein interaction network and functional pathway analysis for intersect genes, GSEA analysis with immune scores as the phenotype. A‐B, We selected partial nodes to establish the interaction network, in which CCL19, TNFSF13B, CD79A, CD19, TNFRSF17 were remarkable nodes. Number of interplay among nodes were calculated in the right barplot. C, Top GO items with *q* < 0.05 were exhibited. D, Top 6 Kyoto Encyclopedia of Genes and Genome (KEGG) pathways were shown with *q* < 0.05. E, Gene set enrichment analysis for comparing phenotype of immune scores between high‐ and low‐levels. A list of 40 immune‐related KEGG pathways enriched with FDR < 0.25 and we selected top 8 in group with high immune scores to display

To deeply understand the underlying interplay among 97 intersect genes, we constructed the PPI network using SRING tool and Cytoscape software. Meanwhile, the number of interactions among nodes was calculated in bar plot (Figure 4B). 111 edges involving 55 genes were formed in the network (Table S3) and we selected some genes to exhibit in Figure 4A, in which CD19, CD79A, TNFSF13B, CCL19 and TNFRSF17 were relatively remarkable nodes. Besides, we further investigated the potential pathways that the 97 genes might be involved in. The GO enriched analysis indicated that these genes may be associated with immune responses, tumor necrosis factors, B cell proliferation, as well as cytokine activity (Figure 4C). Especially, cytokine‐cytokine receptor interaction, hematopoietic cell lineage, and NF‐κB signaling pathway were top significant crosstalk that the 72 genes may participate in (Table 3).

**TABLE 3:** Kyoto Encyclopedia of Genes and Genomes results from the functional pathway analysis of intersect genes

Description	*P* value	*P* adjust	Count
Cytokine‐cytokine receptor interaction	.00000582	.000587719	10
Hematopoietic cell lineage	.000240228	.012131514	5
Primary immunodeficiency	.001278873	.043055382	3
NF‐kappa B signaling pathway	.002240071	.048597712	4
Intestinal immune network for IgA production	.002886993	.048597712	3
Malaria	.002886993	.048597712	3

Since immune scores showed higher associations with overall survival (OS) and other risk clinical variables, we conducted the GSEA analysis to further screen the significant pathway items between groups with higher immune scores against that with lower immune scores. The results revealed that there were a list of 40 gene sets with FDR < 0.25. The top eight immune related pathways included B cell receptor signaling pathway, T cell receptor signaling pathway, Toll‐like receptor signaling pathway, JAK‐STAT signaling pathway, Natural killer cell mediated cytotoxicity, Nod‐like receptor signaling pathway, cytokine‐cytokine receptor interactions, as well as chemokine signaling pathway (Figure 4E).

### 3.4 Batch survival analysis and construction of risk score based on hub genes

**FIGURE 5:**
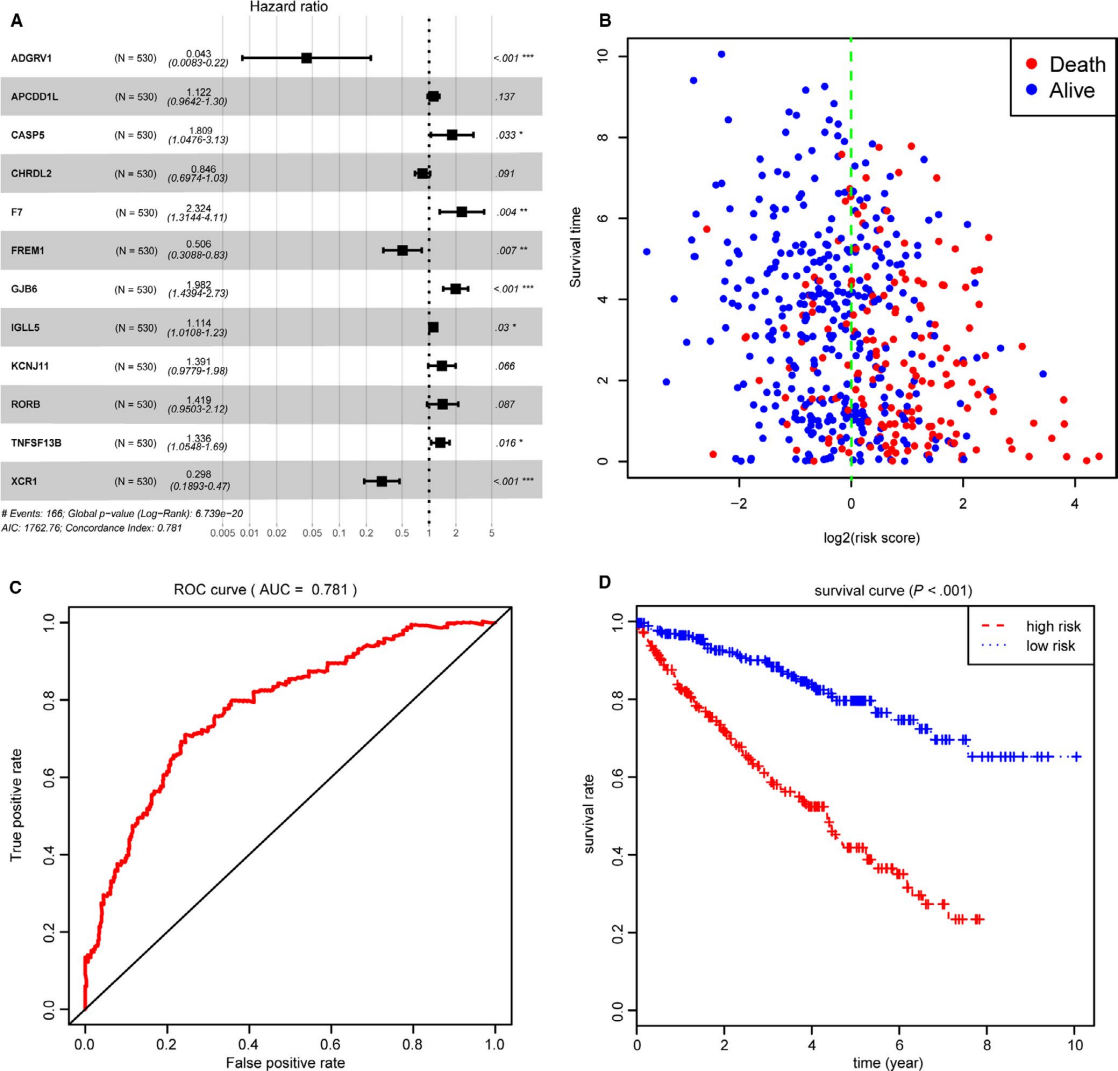
Construction of risk score based on 12 hub genes associated with tumor microenvironment. A, Forest plot of 12 hub genes based on stepwise regression method and multivariate Cox results. B, Distribution of vital status in high‐ and low‐risk groups. C, Receiver Operating Characteristic curve was established for assessing predictive value of risk score with AUC = 0.781. D, Kaplan‐Meier analysis for two levels of risk score indicated that risk score could be an independent risk factor for overall survival in clear cell renal cell carcinoma (*P* < .0001)

We screened 43 prognostic genes with log‐rank test of *P* < .05 from the 72 intersect genes (Table 4). Then, stepwise regression method and multivariate Cox analysis were performed to identify 12 hub prognostic genes associated with ccRCC microenvironment. The risk formula was calculated as: risk score = (−3.14421) * ADGRV1 + 0.11 * APCDD1L + 0.59299 * CASP5‐0.16688 * CHRDL2 + 0.84338*F7 − 0.68058 * FREM1 + 0.68393 * GJB6 + 0.10830 * IGLL5 + 0.32994 * KCNJ11 + 0.34990*RORB + 0.28947 * TNFSF13B − 1.21099 * XCR1 (Figure 5A). Risk score of 530 ccRCC patients was calculated and we got the high‐risk group (n = 265) and low risk group (n = 265) (Table S4). Distribution of vital status of the two risk groups was shown in Figure 5B. Area under the curve (AUC) of ROC for 3‐year OS prediction was 0.781, indicating superior predictive accuracy in survival outcomes. The Kaplan‐Meier plot revealed that ccRCC patients in high‐risk group demonstrated the worse prognosis (Figure 5D). What is more, the survival curves of 12 hub genes with log‐rank test of *P* value were drawn in Figure S6A‐L. The different expressed levels of genes in tumor versus normal were shown in Figure S3.

**TABLE 4:** 43 prognostic genes from the batch survival analysis

Gene name	Description	Location	Log‐rank test of *P* value
PAEP	Progestagen‐associated endometrial protein	Chromosome 9, NC_000009.12	<.001
SLC22A6	solute carrier family 22 member 6	Chromosome 11, NC_000011.10	<.001
OGDHL	oxoglutarate dehydrogenase like	Chromosome 10, NC_000010.11	<.001
GJB6	gap junction protein beta 6	Chromosome 13, NC_000013.11	<.001
SLN	sarcolipin	Chromosome 11, NC_000011.10	0 < .001
OBP2A	Odorant‐binding protein 2A	Chromosome 9, NC_000009.12	<.001
LDHD	lactate dehydrogenase D	Chromosome 16, NC_000016.10	<.001
ADGRV1	adhesion G protein‐coupled receptor V1	Chromosome 5, NC_000005.10	<.001
APCDD1L	APC down‐regulated 1 like	Chromosome 20, NC_000020.11	<.001
SLC22A8	solute carrier family 22 member 8	Chromosome 11, NC_000011.10	<.001
CPA4	carboxypeptidase A4	Chromosome 7, NC_000007.14	<.001
CWH43	Cwh43p	Chromosome III, NC_001135.5	<.001
PPARGC1A	PPARG coactivator 1 alpha	Chromosome 4, NC_000004.12	<.001
HMGCS2	3‐hydroxy‐3‐methylglutaryl‐CoA synthase 2	Chromosome 2, NC_005101.4	<.001
SLC22A12	solute carrier family 22 member 12	Chromosome 11, NC_000011.10	<.001
AQP9	aquaporin 9	Chromosome 15, NC_000015.10	<.001
FDCSP	follicular dendritic cell secreted protein	Chromosome 4, NC_000004.12	<.001
GPAT3	glycerol‐3‐phosphate acyltransferase 3	Chromosome 4, NC_000004.12	<.001
TNFSF13B	TNF superfamily member 13b	Chromosome 13, NC_000013.11	<.001
FREM1	FRAS1‐related extracellular matrix 1	Chromosome 9, NC_000009.12	<.001
HSD11B2	hydroxysteroid 11‐beta dehydrogenase 2	Chromosome 16, NC_000016.10	<.001
MIXL1	Mix1 homeobox‐like 1 (Xenopus laevis)	Chromosome 1, NC_000067.6	.001
FCRL5	Fc receptor like 5	Chromosome 1, NC_000001.11	.001
GREM1	gremlin 1, DAN family BMP antagonist	Chromosome 15, NC_000015.10	.003
MZB1	marginal zone B and B1 cell specific protein	Chromosome 5, NC_000005.10	.004
XCR1	X‐C motif chemokine receptor 1	Chromosome 3, NC_000003.12	.004
ZPLD1	zona pellucida‐like domain containing 1	Chromosome 3, NC_000003.12	.004
CASP5	caspase 5	Chromosome 11, NC_000011.10	.006
TMEM38A	transmembrane protein 38A	Chromosome 8, NC_000074.6	.006
CHRDL2	Chordin‐like 2	Chromosome 11, NC_000011.10	.007
RORB	RAR‐related orphan receptor beta	Chromosome 19, NC_000085.6	.008
IGLL5	immunoglobulin lambda‐like polypeptide 5	Chromosome 22, NC_000022.11	.009
PAH	phenylalanine hydroxylase	Chromosome 12, NC_000012.12	.010
MUC20	mucin 20, cell surface‐associated	Chromosome 3, NC_000003.12	.011
SCARA5	scavenger receptor class A member 5	Chromosome 8, NC_000008.11	.018
KCNJ11	potassium voltage‐gated channel subfamily J member 11	Chromosome 11, NC_000011.10	.019
IL10	interleukin 10	Chromosome 1, NC_000001.11	.027
HSD11B1	hydroxysteroid 11‐beta dehydrogenase 1	Chromosome 1, NC_000001.11	.028
VSIG4	V‐set and immunoglobulin domain containing 4	Chromosome X, NC_000023.11	.029
F7	coagulation factor VII	Chromosome 13, NC_000013.11	.037
RAP1GAP	RAP1 GTPase‐activating protein	Chromosome 1, NC_000001.11	.039
POU2AF1	POU class 2 homeobox‐associating factor 1	Chromosome 11, NC_000011.10	.043
KLK3	Kallikrein‐related peptidase 3	Chromosome 19, NC_000019.10	.044

### 3.5 Validation of hub prognostic genes

**FIGURE 6:**
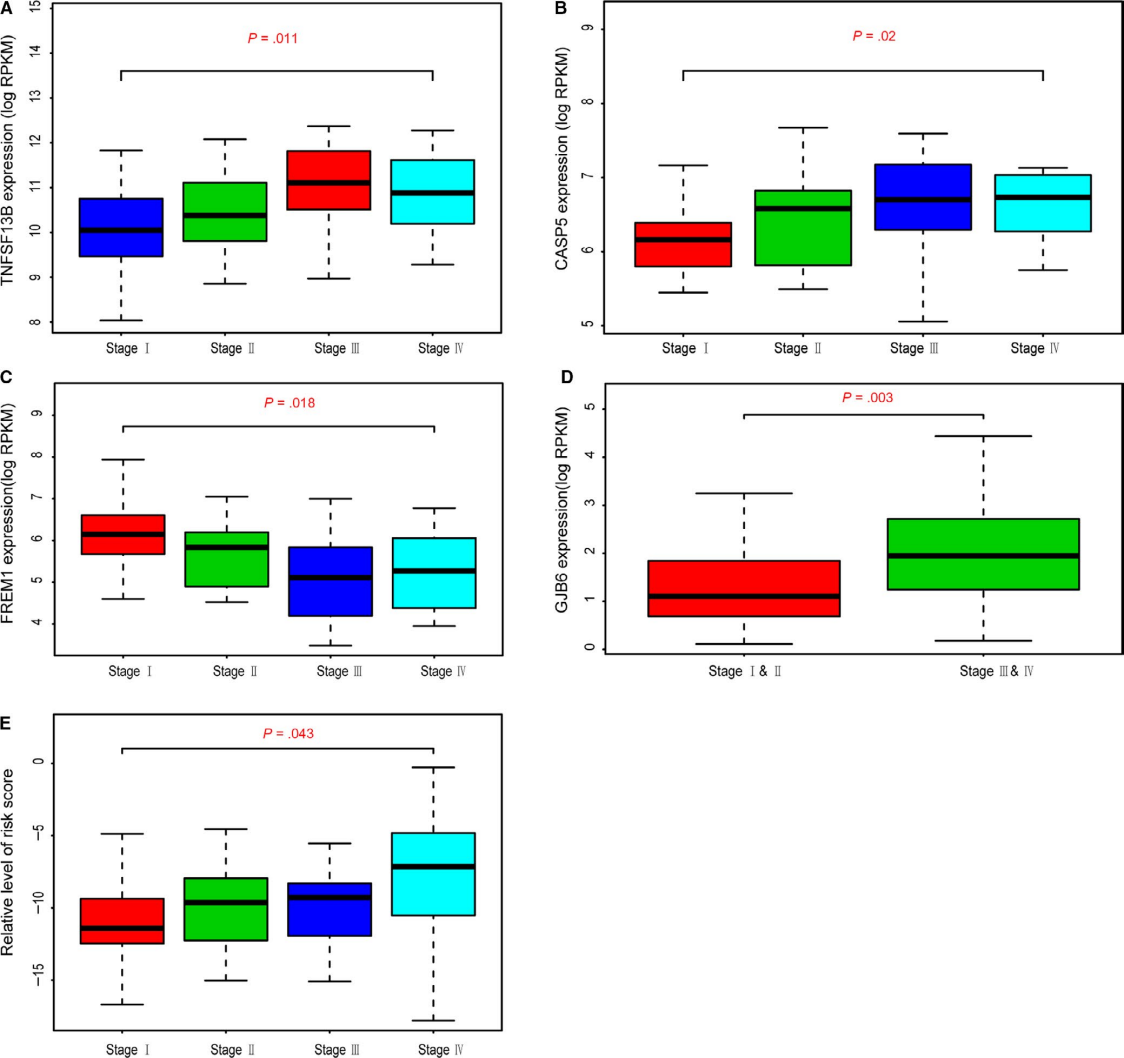
Validation of 12 hub genes in GSE53757. A‐D, Higher expression levels of TNFSF13B, CASP5 and GJB6 correlated higher pathological stages, while level of FREM1 was negatively associated with stages. E, Moreover, risk score calculated as the formula from the TCGA population revealed the same results that higher risk score was related with higher stages (*P* = .043)

To determine whether the 12 hub genes obtained from TCGA cohort remained to be of prognostic significance, we acquired the gene expression profiles of 72 ccRCC patients in an independent data set from GSE53757. We analyzed the gene expression levels of 12 hub genes with clinical tumor stages and found that TNFSF13B, CASP5 and GJB6 correlated positively with tumor stages, while FREM1 showed negative associations with tumor stages. In particular, correlation results in GSE53757 were highly accordant with survival analysis or multivariate Cox analysis in TCGA cohort (Figure 6A‐D). What is more, we calculated the risk score of each sample using the above risk formula and the correlation analysis revealed that patients with higher risk scores showed higher tumor stages with *P* < .05 (Figure 6E).

### 3.6 Correlation analysis between hub genes with immune infiltrates

Since 12 prognostic genes were identified as the hub genes, we attempted to uncover the question how the relationships between hub genes and immune cells infiltration in ccRCC microenrvironmet. Among the 12 genes, five genes were found to be significantly associated with tumor immune infiltrates, where TNFSF13B, CASP5 and XCR1 showed the remarkable correlations with B cell, CD4^＋^ T cell, CD8^＋^ T cell, macrophage, neutrophil and dendritic cell infiltration (Figure S7). Interestingly, TNFSF13B and CASP5 proved to be risk signature in TCGA cohort and correlated with advanced tumor stages in GSE53757. It is worth to implement in‐depth investigations on whether the relationships between expression levels of hub genes and immune infiltrates led to poor survival outcomes in ccRCC microenvironment.

## 4 DISCUSSION

Since immune checkpoint therapies such as nivolumab have developed rapidly in ccRCC in recent years, TME has attracted increasing attention as a crucial cellular milieu incorporating of immune cells, stromal cells as well as extracellular matrix molecules.^29^, ^30^ For example, Toma et al investigated the expression of human 6‐sulfo LacNAc dendritic cells in ccRCC and found that more proportion of 6‐sulfo LacNAc dendritic cells was negatively associated with progression‐free, tumor‐specific or overall survival.^31^ In addition, human endogenous retroviruses sequences were identified significantly overexpressed in ccRCC tumors with sensitivity to programmed death receptor 1 (PD‐1) inhibition therapy.^32^ Unlike most studies that focused on a nontumor cell or immune molecule in TME, our current study was based on certain high‐quality datasets, and identified specific signatures that related to the infiltration of stromal and immune cells in ccRCC TME by using algorithm that takes advantage of the transcriptional profiles.

Recently, there have been more and more applications of bioinformatics in the field of medical research.^33^, ^34^ ESTIMATE algorithm was presented by Yoshihara in 2013 at first time.^17^ In glioblastoma, ESTIMATE algorithm‐derived immune scores and stromal scores were performed to facilitate the quantification of the non‐tumor components in a malignancy.^9^ In our current research, we calculated the immune scores, stromal scores and ESTIMATE scores for each ccRCC sample extracted from the TCGA database by applying ESTIMATE algorithm. The results revealed that immune scores were statistically significantly higher in malignant tumor cases and associated with worse survival outcomes, higher AJCC‐T level, higher AJCC‐N level, higher AJCC‐M level, advanced tumor grades and higher pathological stages. For the first time that ESTIMATE algorithm‐derived immune scores were calculated in ccRCC to evaluate the prognostic value and provide extra evidence for the biological basis of immunotherapy.

In our study, PPI network was constructed using SRING tool and Cytoscape software. Relatively remarkable nodes including CD19, CD79A, TNFSF13B, CCL19 and TNFRSF17 were selected and the potential pathways such as cytokine‐cytokine receptor interaction, hematopoietic cell lineage, and NF‐κB signaling pathway were identified by GO enriched analysis. It was reported that a potential pathologic p.G76S heterozygous mutation on the TNFRSF13B gene which identified by whole‐exome sequencing might upregulate cytokine‐cytokine receptor interaction signaling pathway and increase serum TNFα, IL‐17α, IFNγ and BAFF levels in immune thrombocytopenia patients.^35^ In addition, CD19 was revealed to participate in the regulation of constitutive activation of NFκB pathway in chronic lymphocytic leukemia as a role of hematopoietic cell lineage marker.^36^ Apart from classical NFκB pathway, Wharry et al found that CCL19 was dramatically elevated in pancreatic cancer cells acting as noncanonical NFκB target gene.^37^ However, relevance of above remarkable nodes genes and pathways in ccRCC require further investigation.

Finally, a total of 12 hub prognostic genes associated with TME were identified by stepwise regression method in multivariate Cox analysis. We explored the associations between hub genes with B cell, CD4^＋^T cell, CD8^＋^T cell, macrophage, neutrophil and dendritic cell infiltration analyzed by using the deconvolution algorithm based on the TIMER database. Furthermore, TNFSF13B and CASP5 were proved to be correlated with advanced tumor stages in GSE53757.

Tumor necrosis factor ligand superfamily member 13B (TNFSF13B) also known as B‐cell activating factor (BAFF) is a cytokine that belongs to the tumor necrosis factor (TNF) ligand family. As a potent B cell activator, TNFSF13B is identified in the biological process of B cell proliferation and differentiation.^38^ Previous studies on TNFSF13B have mostly focused on immune system diseases and hematological malignancies. Current researches indicated that TNFSF13B, which might be significantly affected by IFN regulatory factors,^39^, ^40^ is an important regulatory target in primary Sjögren's syndrome (SS). Ding et al reported that overexpressed TNFSF13B might increases lymphocytic infiltration and inefficiently promotes ectopic B‐cell differentiation in SS.^41^ Besides, studies revealed that genetic variants of both TNFSF13B and TNFSF13B‐receptor were related to SS‐related lymphoma.^42^, ^43^, ^44^ In contrast with our research, Pelekanou et al observed a differential expression of TNFSF13B in 86 ccRCC tissues detected by immunohistochemistry, while independent of tumor grade.^45^ Compared to our genome‐wide bioinformatics analysis based on multiple‐database, the conflicting result may be caused by the more significant bias from single‐center small sample‐sized study. It is noticeable that limited research focused on specific regulation mechanism for the role of TNFSF13B in ccRCC, and the evidence we provide in terms of immune infiltration may serve as a potential research strategy.

Caspase 5 (CASP5), along with CASP1, CASP4, and CASP12, belong to inflammatory caspases sub‐family, which were identified to play a role in the maturation of inflammatory cytokines (IL‐1β and IL‐18) and apoptosis pathways.^46^, ^47^, ^48^ In human monocytes, CASP5 and CASP4 could be activated by saturated fatty acids, then trigger IL‐1β and IL‐18 release, which contributed to type 2 diabetes.^49^ Apart from regulating obesity‐associated inflammation, CASP5 might be particularly important for carcinogenesis. Dong et al identified rs507879, which was located within exon 2 of CASP5 and resulted in a missense mutation and amino acid substitution.^50^ Although this CASP5 exon 2 SNP is discovered to be a benign mutation by PolyPhen. However, a common somatic mutation in exon 2 was observed in leukemias and some malignant solid tumors including gastric, colon, and lung cancers yy.^51^, ^52^, ^53^, ^54^ According to our results, CASP5 was firstly proved to be correlated with advanced tumor stages of ccRCC in GSE53757. Combined with the above findings, inflammatory cytokines‐derived apoptosis pathway might be a possible mechanism.

Remarkably, the risk model was calculated based on 12 hub prognostic genes associated with TME of ccRCC. The AUC of the ROC curve revealed the satisfactory predictive efficiency of the risk signature. After that, we validated the prognosis value of the risk model in an independent data set from GSE53757. This novel TME hub genes‐related risk score model provides a new theoretical basis for the prognosis assessment of ccRCC patients, which is expected to be further applied in the future clinical management.

Of note, there still exists several limitations in the current study. Firstly, we only selected sequencing data from public databases analyzed through biological algorithm approaches. We should validate the results from this article in clinical patients, which was warranted in our own cohorts. Secondly, 12 TME‐related hub genes should be further studied to clarify the regulatory mechanism in immune infiltrates of ccRCC. Finally, considering the choice of analytical approaches, we included a limited database for the screening of hub genes, which may result in biased results due to the neglect of other databases.

In summary, a list of TME‐related hub genes was extracted from functional enrichment analysis of TCGA database based on ESTIMATE algorithm. After survival analysis and prognostic value evaluation, these hub genes might become potential biomarkers of ccRCC. Besides, risk score which was calculated based on hub genes provided a new theoretical basis for predicting survival conditions of ccRCC patients. Finally, we further shed the insights on the potential associations of TME‐related signature with tumor immune‐infiltrating abundance.

## 5 CONCLUSION

In our research, we selected the transcriptional profiles from public databases based on bioinformatics algorithm and identified specific signatures that related to the infiltrating levels of stromal and immune cells in TME of ccRCC. Overall, our research could provide a comprehensive understanding of tumor microenvironment and potential foundations for future individualized therapy.

## CONFLICT OF INTEREST

The authors declare that the research was conducted in the absence of any commercial or financial relationships that could be construed as a potential conflict of interest.

## AUTHOR CONTRIBUTIONS

Yaoting Xu and Jun Luo designed the study and analyzed the data; Jun Luo and Yi Xie drafted the article; Yuxiao Zheng, Chenji Wang, Qi Feng, and Jiateng Hu were responsible for language correction. All authors finally approved the paper.

## Supporting information

Fig S1

Fig S2

Fig S3

Fig S4

Fig S5

Fig S6

Fig S7

Table S1

Table S2

Table S3

Table S4

## Data Availability

The data that support the findings of this study are available from the corresponding author upon reasonable request.
