# Supplementary figures and images for: Comprehensive insights on pivotal prognostic signature involved in clear cell renal cell carcinoma microenvironment using the ESTIMATE algorithm
Source: Cancer Med. 2020 Apr 20;9(12):4310–23. doi: 10.1002/cam4.2983 (PMC7300420; doi:10.1002/cam4.2983)

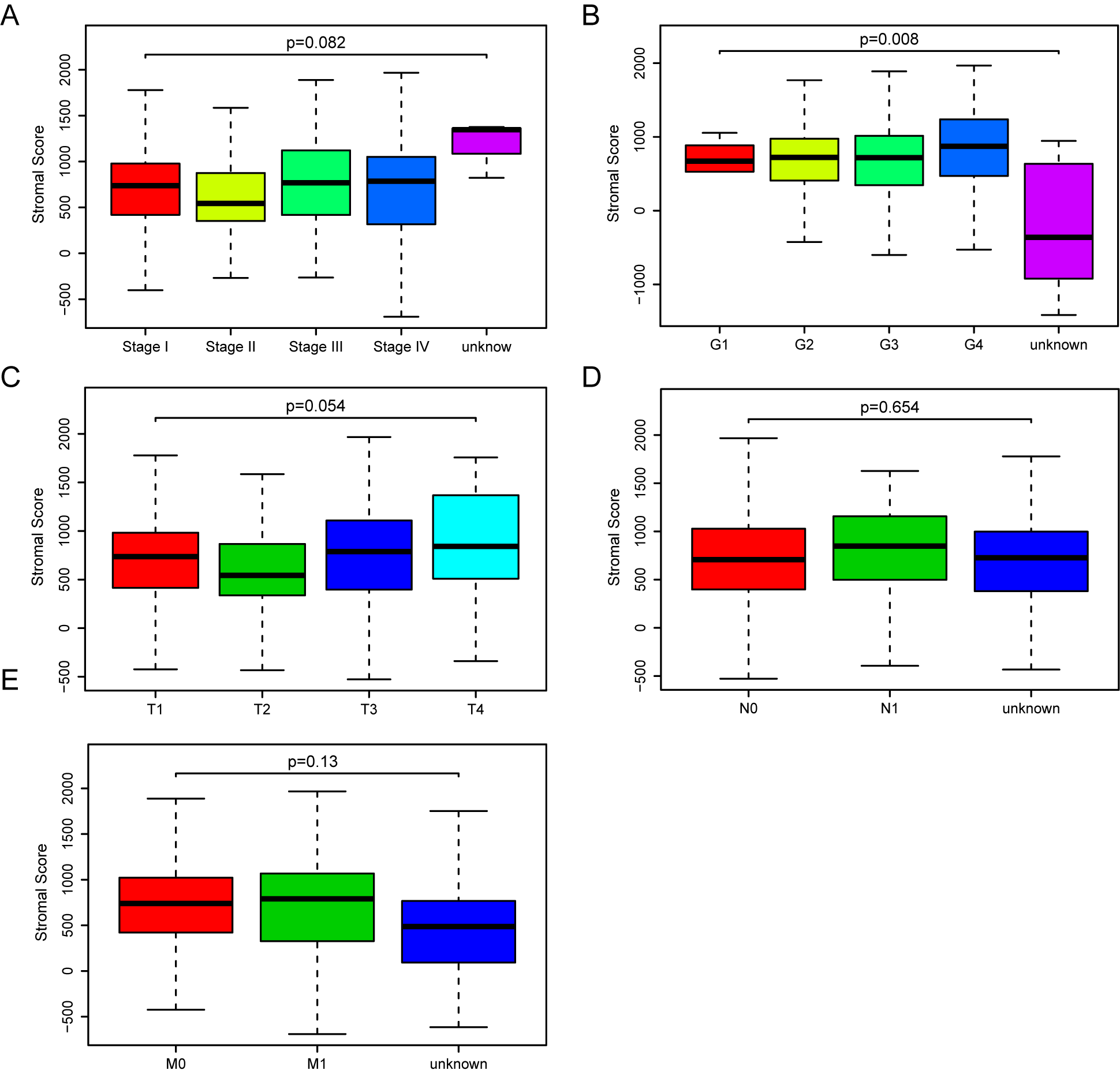

Supplement: Supplementary file 1 — Fig S1 [file CAM4-9-4310-s001.tif]

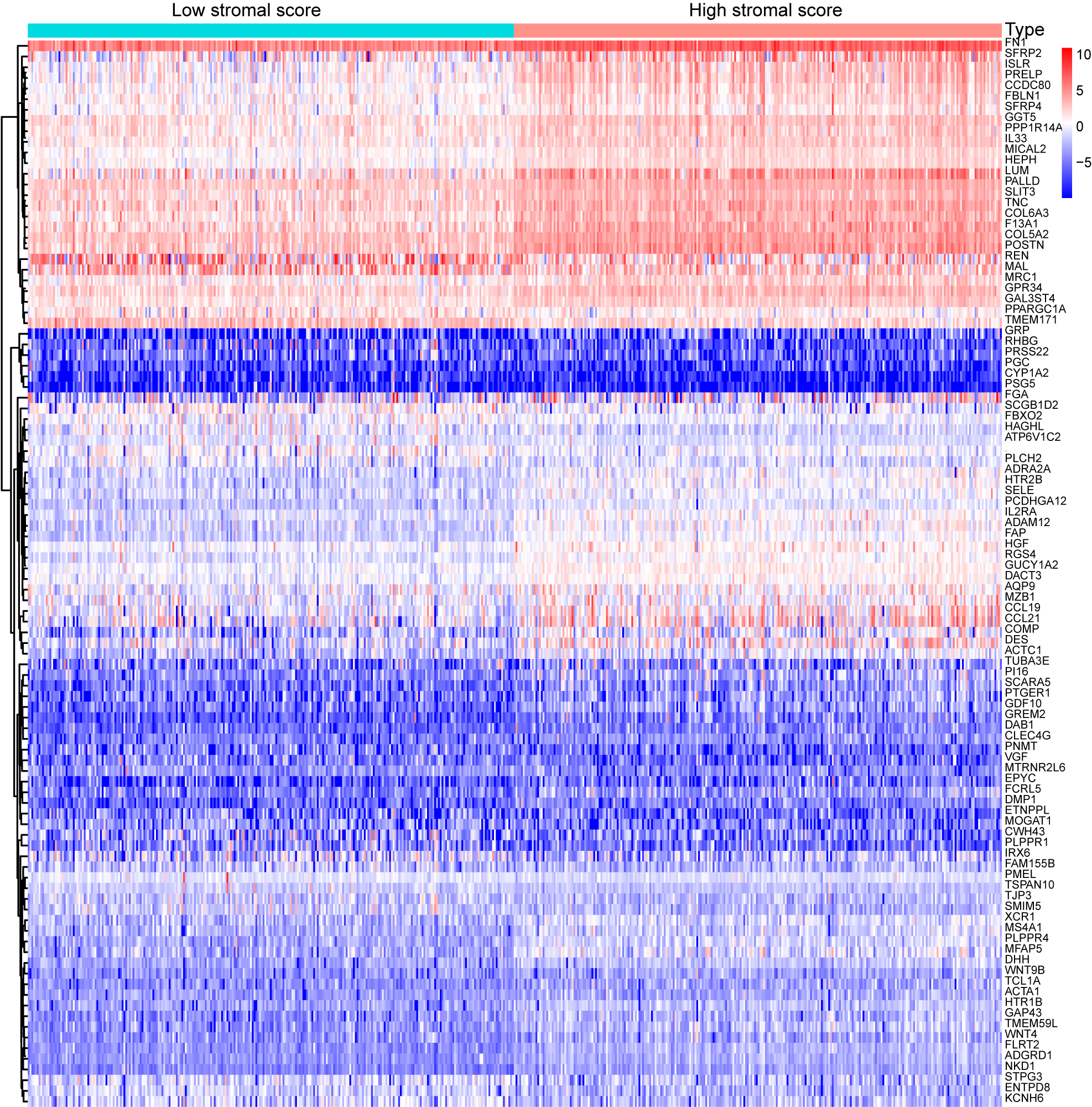

Supplement: Supplementary file 2 — Fig S2 [file CAM4-9-4310-s002.tif]

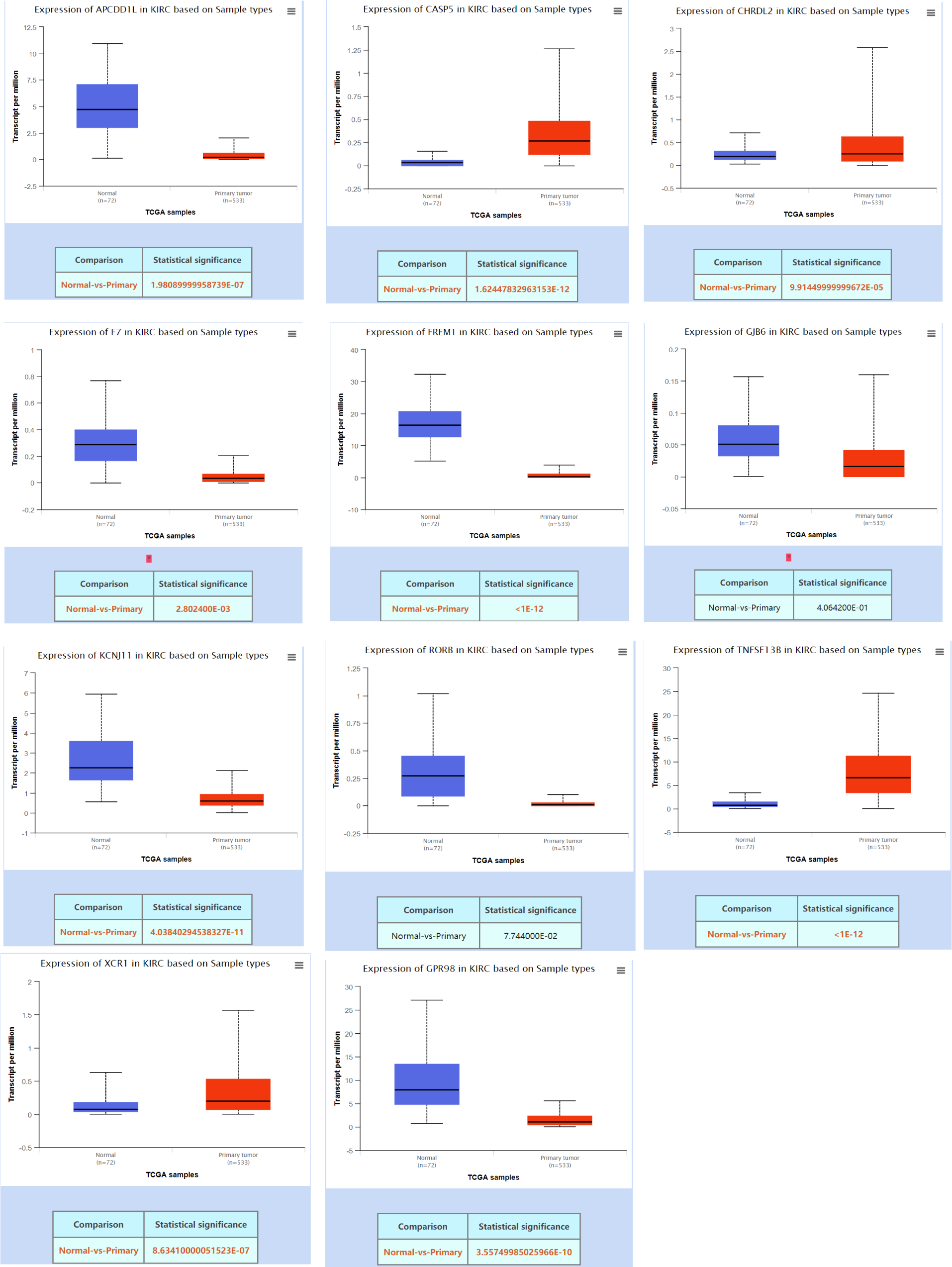

Supplement: Supplementary file 3 — Fig S3 [file CAM4-9-4310-s003.tif]

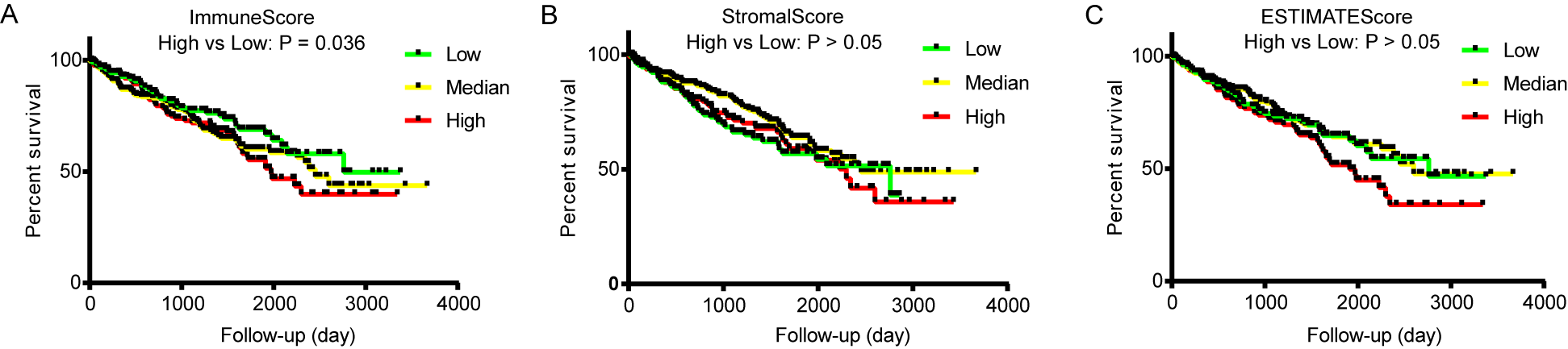

Supplement: Supplementary file 4 — Fig S4 [file CAM4-9-4310-s004.tif]

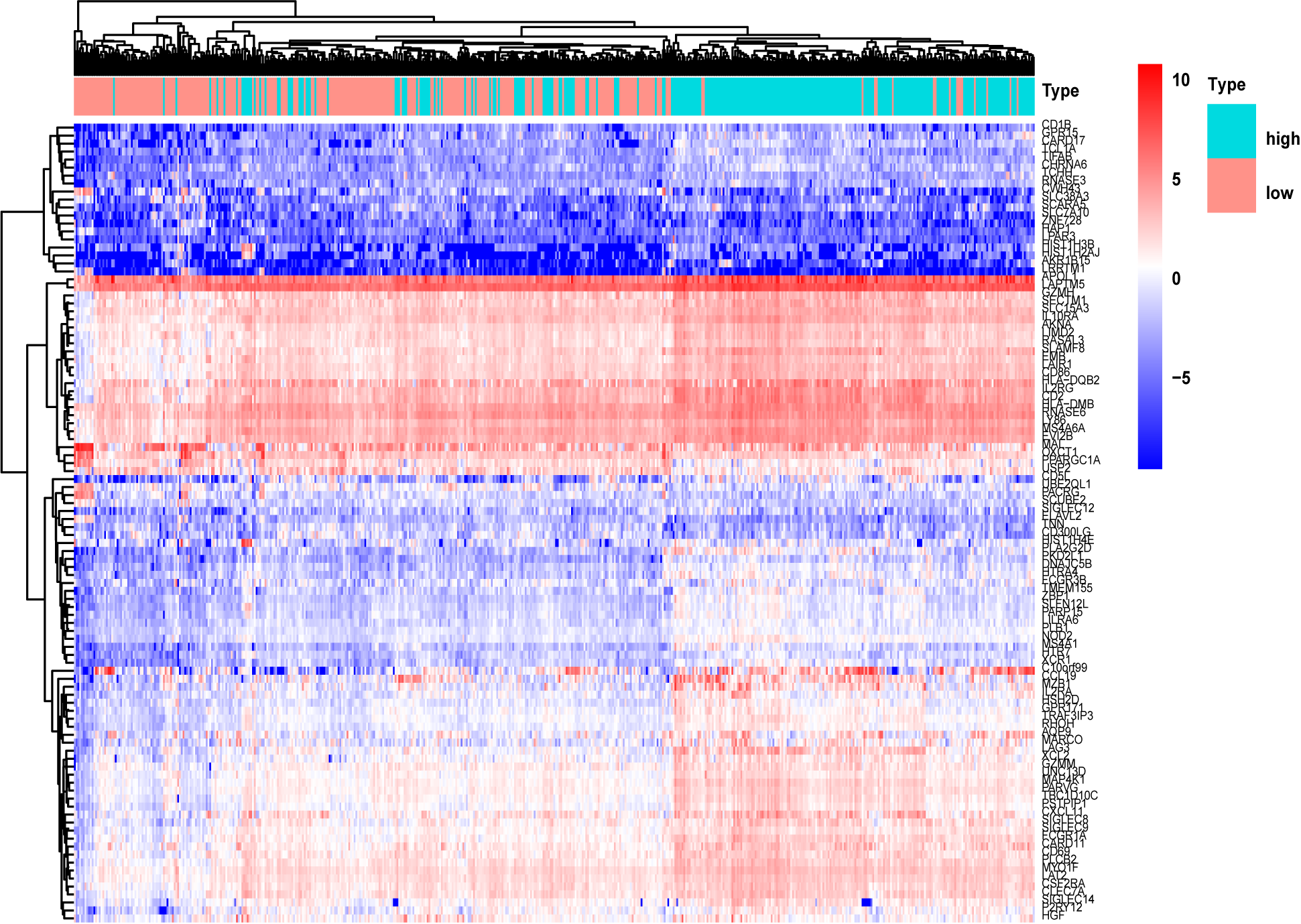

Supplement: Supplementary file 5 — Fig S5 [file CAM4-9-4310-s005.tif]

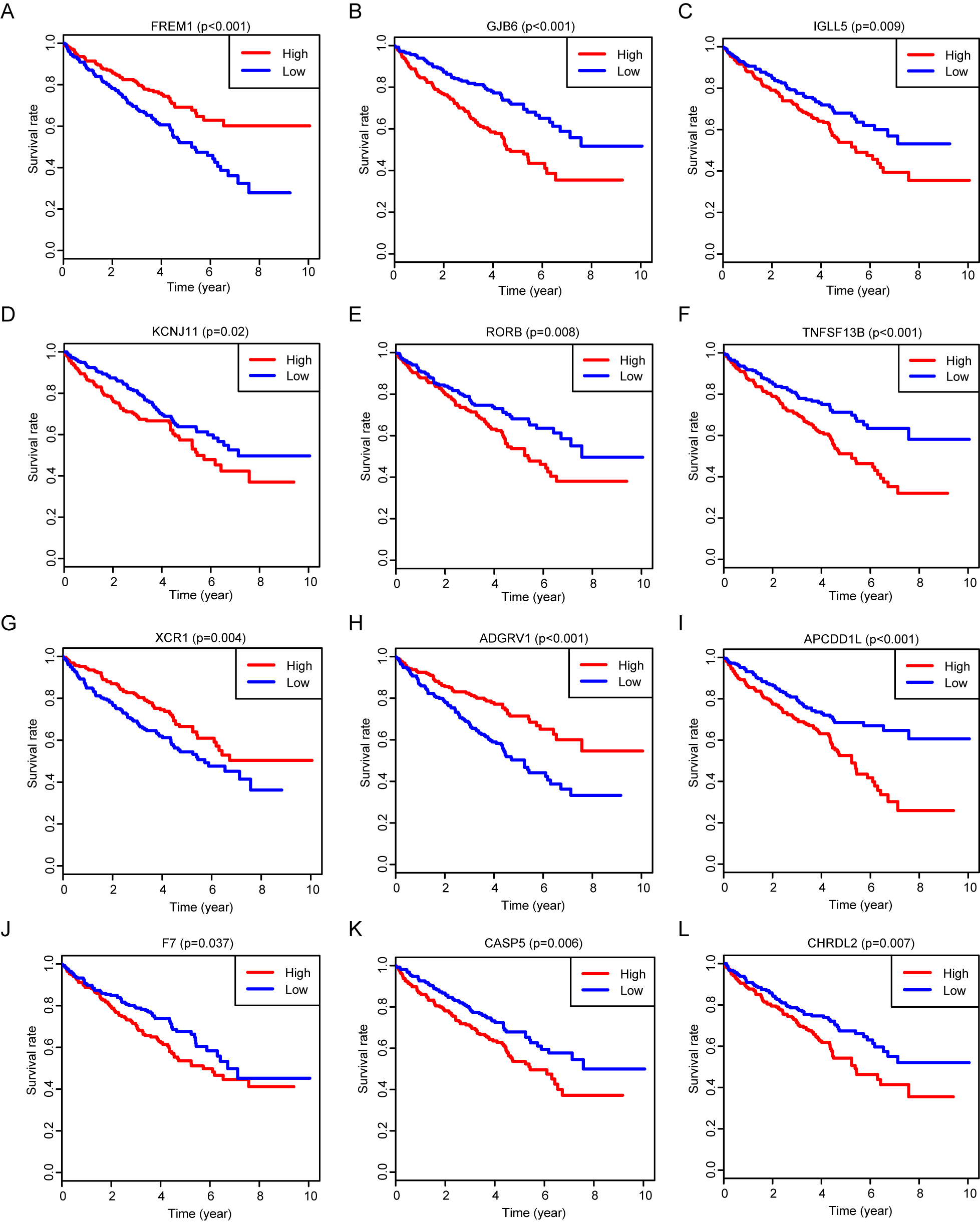

Supplement: Supplementary file 6 — Fig S6 [file CAM4-9-4310-s006.tif]

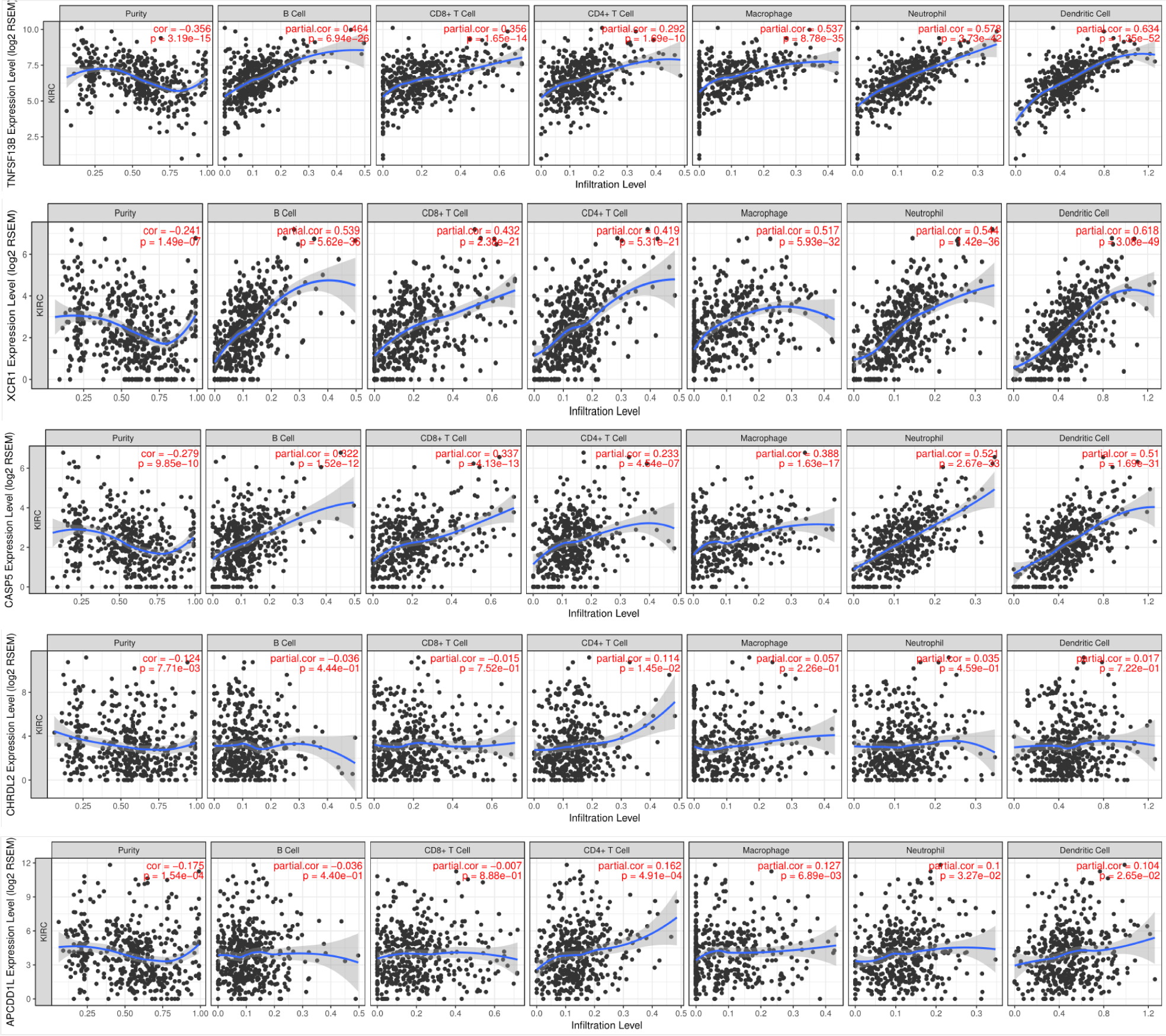

Supplement: Supplementary file 7 — Fig S7 [file CAM4-9-4310-s007.tif]
